# Population-based cohort study to investigate the changes in prevalence, severity profile, and treatment modalities used in Korean atopic dermatitis patients

**DOI:** 10.1038/s41598-024-57777-6

**Published:** 2024-04-05

**Authors:** Hyun Ji Lee, Hyun Ju Oh, Gyu Na Lee, Kyung Do Han, Ji Hyun Lee, Young Min Park

**Affiliations:** 1 Department of Dermatology, Yeouido St. Mary’s Hospital, Seoul, Korea; 2 Department of Dermatology, Seoul St. Mary’s Hospital, College of Medicine, The Catholic University of Korea, 222 Banpo-daero, Seocho-gu, Seoul, 06591 Korea; 3 Department of Statistics and Actuarial Science, Soongsil University, Seoul, Korea

**Keywords:** Atopic dermatitis, Epidemiological study, Eczema, Treatment, Skin diseases, Epidemiology

## Abstract

In this retrospective study spanning from 2002 to 2019, we analyzed data from 355,277 Korean patients diagnosed with atopic dermatitis (AD) through the National Health Insurance System. Our objective was to comprehensively analyze the trends in prevalence, severity profiles, and treatment approaches for AD in Korea over this 18-year period. Initially, AD prevalence stood at 3.88% in 2002 but notably rose to 5.03% by 2019. During the same period, while AD prevalence decreased in the 0–1-year-old group (from 34.52% to 24.83%), it remained relatively stable in the 1–11-year-old group. Conversely, the 12–19-year-old and 20 years or older age groups witnessed substantial increases in AD prevalence, climbing from 2.55 to 6.02% and 1.44% to 3.53%, respectively. Moreover, the proportion of patients classified as having moderate to severe AD grew from 30.96 to 39.78%. Surprisingly, the prescription pattern, predominantly based on corticosteroid administration, exhibited minimal change despite the rising prevalence of moderate and severe AD cases. These findings underline a persistent reliance on corticosteroid-based treatments for AD, even as the condition's severity escalates among Korean adolescents and adults. Consequently, there is a pressing need to develop novel treatment guidelines emphasizing biologics that offer enhanced safety and efficacy.

## Introduction

Atopic dermatitis (AD) is a chronic inflammatory skin disease that has a profound impact on quality of life^1^. The prevalence of AD is highest in infants and tends to decrease with age^2,3^ It is now estimated to affect 8–20% of children and 2–17% of adults, showing heterogeneous results depending on country and research method^4–9^. In a few studies that have been performed in Korea, the prevalence of AD tends to decrease in children but increase in adults. Thus, it is necessary to observe recent changes according to age. Although it is difficult to clearly compare the relative percentages of the patient group classified as moderate to severe due to differences in definition among studies, approximately 20–30% were classified as moderate and 10% as severe disease in Japan and USA^6,7,9–12^. In Korea, 47.1% of patients were reported as having moderate disease, while 10.9% had severe disease^10^.

The primary treatment goal for AD is to improve symptoms and establish long-term disease control. Avoidance of individual triggers, restoration of the skin barrier using moisturizers, and use of anti-inflammatory agents according to severity of disease are the basic framework of treatment of AD^11,12^. Mild cases of AD can usually be controlled with topical treatments, but more severe disease status could require not only systemic immunosuppressants including cyclosporine, corticosteroid, methotrexate, and azathioprine, but also phototherapy^13,14^. Recently, due to the side effects of immunosuppressants, several biologics have been developed and used in clinical practice. Dupilumab, a monoclonal antibody blocking interleukin 4 and interleukin 13, and Janus kinase inhibitors have been effectively used to treat severe AD^15^.

Although there are various treatment options for moderate to severe AD, analysis of real-world treatment patterns for the Korean population is lacking. Recent large-scale studies using a national database in Korea have focused on the prevalence of AD^16,17^.

In this study, we aimed to comprehensively analyze the trends in prevalence, severity profiles, and treatment approaches for AD among the Korean population over an 18-year period from 2002 to 2019.

## Materials and methods

### Study design and data source

This retrospective, observational study was performed using data collected from the National Health Insurance Service–National Sample Cohort (NHIS-NSC) database in Korea from January 1, 2002, to December 31, 2019. This database includes a complete set of eligibility criteria, claims for medical expenses, and health screening data.

All methods were carried out in accordance with relevant guidelines and regulations. Informed consent was not obtained for the individual human participants since this was a retrospective study using the NHIS claims database, not an experimental study including human subjects. Data used from the Korea National Health Insurance Service (NHIS) claims database is a publicly accessible, upon reasonable request to and with the permission of NHIS. This study was approved by the Institutional Review Board of The Catholic University of Korea (No. KC20ZISI0441) and used data (NHIS REQ202200755-002) made available by the NHIS. Informed consent requirement was waived by the same ethics committee, Institutional Review Board of The Catholic University of Korea (No. KC20ZISI0441) for this retrospective study.

### Study population and definition of AD

Subjects who maintained their national health insurance or medical care status between January 1, 2002, and December 31, 2019 were included. Subjects aged < 18 years and those with missing data were excluded. We identified all patients who were diagnosed with AD and subsequently received at least one prescription with code L20 (International Classification of Diseases-10th Revision). In our study, we defined AD patients as those who had ever billed insurance with the code L20, and the annual prevalence of AD was defined as the number of patients divided by the estimated population in each year. After stratifying total AD patients into 4 groups by age (0–1 year, 2–11 years, 12–19 years, and 20 years or older), we compared the annual prevalence of AD among groups from 2002 to 2019. Data regarding the estimated population of all age groups from 2002 to 2019 were collected from Statistics Korea (http://www.kostat.go.kr).

### Treatment patterns and proxy for severity of AD

We collected information on prescriptions in the study population from 2002 to 2019. We extracted information on the numbers of prescriptions for systemic antihistamines, corticosteroid, and other immunosuppressants (cyclosporine, azathioprine, methotrexate, mycophenolate mofetil) and of phototherapy by year. The prescription codes of topical agents have changed since 2016, so those who were prescribed only topical agents before 2016 were not included in the AD patient group. We defined patients who had been treated with systemic agents including corticosteroid, immunosuppressants, and phototherapy as the moderate to severe group.

## Results

### Annual prevalence

**Figure 1:**
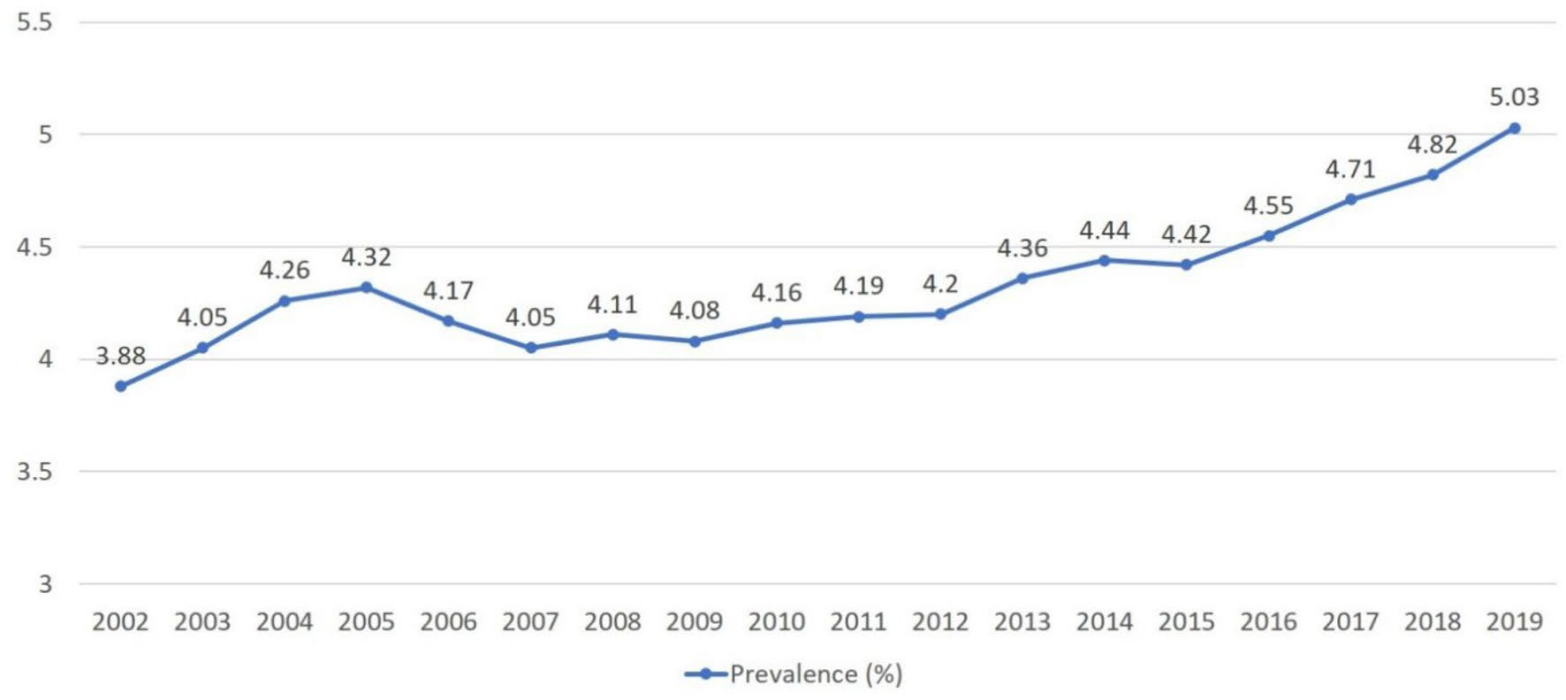
Change of annual prevalence of atopic dermatitis in Korea from 2002 to 2019.

**Figure 2:**
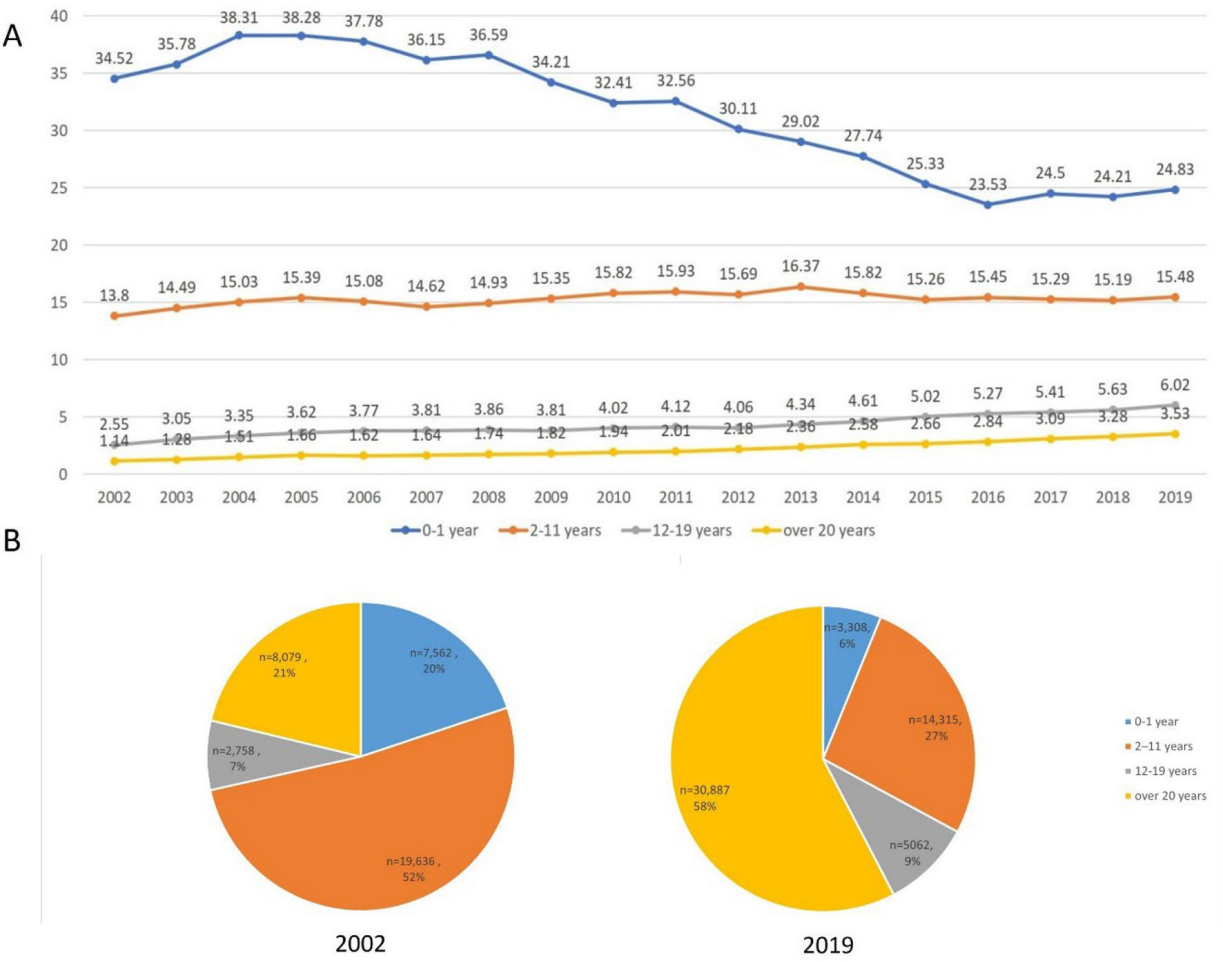
(**A**) Change of annual prevalence of atopic dermatitis according to age group from 2002 to 2019, (**B**) distribution of atopic dermatitis patients by age group in 2002 and in 2019.

Figure 1 shows the annual prevalence of AD from 2002 to 2019. The prevalence of AD in total patients enrolled, which was 3.88% in 2002, increased to 5.03% in 2019. Figure 2A showsthe prevalence of AD by age group. In 2002 the annual prevalence of AD in the 0–1 year group, 2–11 years group, 12–19 years group and 20 years or older group was 34.52%, 13.5%, 2.55%, and 1.44%, whereas in 2019 it was 24.83%, 15.5%, 6.02% and 3.53%. Similarly, the relative proportions of AD patients in these age groups showed 20%, 52%, 7%, and 21% in 2002, which changed to 5%, 27%, 9%, and 58% in 2019, respectively (Fig. 2B).

### Severity profile

**Figure 3:**
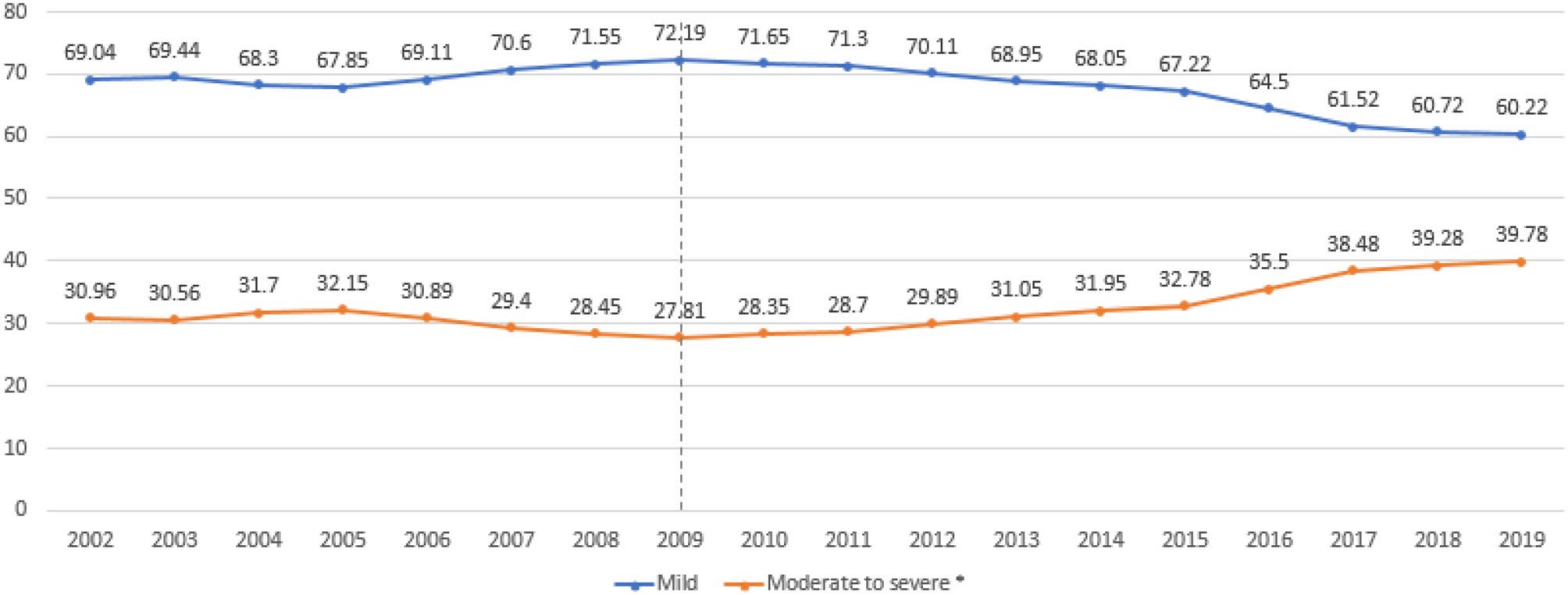
Change of annual relative percentage of patients with mild vs. moderate or severe atopic dermatitis from 2002 to 2019.

The moderate to severe group represented 29.82% of total AD patients throughout the study period (Table 1). Relative proportion by age group was 8.19% in the 0–1 year group, 24.82% in the 2–12 year group, 43.97% in the 12–19 year group, and 41.87% in the 20-year-old or older group. From 2002 to 2019, the percentage of patients in the moderate to severe group increased from 30.96 to 39.78%, and especially increased after 2009 (Fig. 3).

**Table 1:** Proxy for AD severity: moderate to severe: at least 1 prescription claim of systemic corticosteroid, systemic immunosuppressants, or phototherapy.

Age group	Total	0–1 year	2–12 years	12–19 years	≥ 20 years
Total number of patients	355,277	85,995	83,735	20,507	165,040
Mild (%)	249,336 (70.18)	78,955 (91.81)	62,951 (75.18)	11,491 (56.03)	95,939 (58.13)
Moderate to severe (%)	105,941 (29.82)	7040 (8.19)	20,784 (24.82)	9016 (43.97)	69,101 (41.87)

### Treatment patterns

The number of yearly hospital visits per AD patient was 1.83 ± 1.56 in 2002 and 2.03 ± 3.05 in 2019. Table 2 shows the number of prescriptions for each drug by year. Prescription percentage of systemic antihistamines accounted for 38.87% in 2002 and 48.37% in 2019. Prescription percentage of systemic corticosteroid was 30.72% in 2002 and 38.65% in 2019. Those of immunosuppressants and phototherapy were 0.45% and 0.08% in 2002 and 1.71% and 1.56% in 2019.

**Table 2:** Treatment patterns of atopic dermatitis in Korea from 2002 to 2019.

Year	2002	2005	2008	2011	2014	2017	2019
Total number of patients	38,035	43,671	42,556	43,865	47,001	50,190	53,572
Visits per year	1.83 ± 1.56	1.5 ± 1.1	1.94 ± 2.24	2 ± 2.98	1.94 ± 2.41	2.02 ± 3.03	2.03 ± 3.05
Systemic treatments (%)							
Antihistamines	14,783 (38.87)	17,009 (38.95)	17,273 (40.59)	17,659 (40.26)	19,640 (41.79)	25,850 (51.5)	25,911 (48.37)
Corticosteroid	11,683 (30.72)	13,889 (31.8)	11,931 (28.04)	12,346 (28.15)	14,655 (31.18)	18,871 (37.6)	20,705 (38.65)
Immunosuppressants							
Azathioprine	1 (0)	4 (0.01)	5 (0.01)	16 (0.04)	13 (0.03)	45 (0.09)	45 (0.08)
Cyclosporine A	29 (0.08)	41 (0.09)	80 (0.19)	189 (0.43)	346 (0.74)	531 (1.06)	712 (1.33)
Methotrexate	1 (0)	5 (0.01)	5 (0.01)	18 (0.04)	18 (0.04)	34 (0.07)	77 (0.14)
MMF	0 (0)	0 (0)	8 (0.02)	4 (0.01)	7 (0.01)	10 (0.02)	13 (0.02)
Phototherapy							
1–11 times	169 (0.44)	318 (0.73)	262 (0.62)	255 (0.58)	421 (0.9)	602 (1.2)	777 (1.45)
≥ 12 times	2 (0.01)	4 (0.01)	23 (0.05)	29 (0.07)	71 (0.15)	94 (0.19)	139 (0.26)

*MMF* mycophenolate mofetil.

## Discussion

This study is meaningful in that it analyzed trends in AD over a long period of 18 years from 2002 to 2019. Based on our results, the overall prevalence of AD increased gradually over this time. In addition, in the analysis by age group, the prevalence of infants aged 0–1 decreased, but the prevalence of adults increased considerably. In 2002, the 2–11 years group accounted for the largest number of AD patients, whereas in 2019, the 20 years or older patient group accounted for the largest number. The proportion of patients in the moderate to severe group has steadily increased, especially since 2009. For analysis of prescription pattern, the proportions of systemic antihistamines and corticosteroid showed little change during the study period. Although their absolute numbers were small, the numbers of cyclosporine and phototherapy prescriptions increased significantly in 2019 compared to 2002. This might be a result of the relatively increased proportion of moderate to severe AD patients in adolescence and adulthood.

Our study showed that the prevalence of AD in infants and children decreased, while the prevalence in adults increased. According to previous studies, the prevalence of AD in adults was about 7% in the United States in 2019^6^ and varied internationally from 2.0 to 17.6%^18^. In Korea, the prevalence of AD in adults was reported to be 2.6% in 2010 and 3.9% in 2020^3,17^. Ha et al.^17^ studied AD prevalence in Korea by age group from 2008 to 2017 and showed a significant increase in prevalence in the group aged 60 years or older. In another study conducted by Kim et al.^2^, there was no significant change in the prevalence of AD in children aged 10 years or older from 2009 to 2014, during which the prevalence of AD in the total sample decreased by 2.6% per year. Other studies have reported that the prevalence of AD in children younger than 10 years is gradually decreasing in Korea^17,19,20^, which was consistent with our result. Considering reports of decreasing or constant trend in prevalence of AD in children except in developing countries, the increase in AD prevalence in our study can be attributed to an increase in adults. Airborne pollution, increased socioeconomic status, psychological stress, and health care utilization are possible causes of the increase in prevalence of AD in adults^18^.

The proportion of patients with moderate to severe AD showed a steady increase from 2009 to 2019 in our study. Internationally, the proportion of patients with moderate to severe AD among total patients is 20% to 37% for moderate AD and 10% to 34% for severe AD^21–23^. A small number of studies on the prevalence of moderate to severe AD in Korea has been reported. In children, moderate to severe AD has been reported in 1.5% to 6.5% of cases^16,24,25^. A recent study reported moderate to severe disease in 4.2% of adult AD patients^24^. The large variation in proportion can be attributed to differences in population, race, and medical environment, in addition to lack of a clear definition of moderate to severe AD.

The treatment guidelines for AD in Korea published in 2021 were as follows. The first drug to be considered for treatment of moderate to severe AD is cyclosporine, and azathioprine, methotrexate, and MMF are recommended as second-line drugs. Systemic corticosteroid are only recommended for acute usage based on concerns about their side effects and rebound phenomenon. Dupilumab is recommended for patients with moderate to severe AD^26,27^. In our study, immunosuppressants other than corticosteroid accounted for only 5% of the total prescription. Considering the steady increase in moderate to severe AD and the increase in prevalence of AD in adults who have relatively little concern about the side effects of immunosuppressants, the small percentage of prescriptions for immunosuppressants suggests that appropriate treatment is not being provided to moderate to severe AD patients. This highlights the need for treatment guidelines that include novel drugs, such as dupilumab, baricitinib, abrocitinib, and delgocitinib, which have been recently spotlighted as safe and effective treatments for AD.^27^

This study has several limitations. First, our definition of AD patients as those who received prescriptions at least once may not accurately reflect the actual AD patient population as it excluded those who did not receive any prescription or who did not visit a hospital. Second, as a large-scale cohort study, analysis according to the diagnostic criteria for AD could not be performed. The database we used does not contain information about symptoms and signs. Hence, we should have set the proxy for severity based on prescriptions rather than a clinical scoring system such as Eczema Area and Severity Index(EASI).^28^ In very young children, topical treatment is given instead of systemic treatment, even though the eczema is moderate to severe. This could have distorted our calculated proportion of moderate to severe eczema especially at this age group. Third, those who were prescribed only topical agents before 2016 were not included in the AD patient group, which may have distorted the results, as the AD population has increased since 2016. Also, the use of biologics and the newer JAK inhibitors may not be reflected in the database as in the years before 2019, the use of these treatments were not so prevalent. And even after becoming available, the use of these medications could have been limited due to the expensive price. Lastly, as it was a National Health Insurance Service data-based study, we do not exactly know which specialist diagnosed the patient with AD. Therefore, we were unable to separate the analysis between patients diagnosed with AD by dermatologists and other specialists who could have affected an accuracy of diagnosis. Detailed information on the strength of topical steroids prescribed was also unavailable, hindering the opportunity to define the severity of eczema more accurately. Nevertheless, our study is significant in that it analyzed the trends in AD prevalence, proportion of moderate to severe cases, and prescription patterns using a database representing the entire population of Korea over a long period of nearly 2 decades.

## Conclusion

These results demonstrate little change in the corticosteroid-based treatment pattern despite the increase in prevalence of moderate or severe AD in Korean adolescents and adults over the past 18 years. Therefore, it is necessary to establish new drug-oriented treatment guidelines including biologics to achieve improved safety and effectiveness in treatment of AD.

## Data Availability

National Health Insurance data used in this study are available from the Korea National Health Insurance Service (NHIS) under restricted access due to data protection legislation and are available upon reasonable request to and with the permission of NHIS (https://www.nhis.or.kr/). First author (Hyun Ji Lee) is the one to contact if anyone wants to request the data from this study.
